# Be Aware Not Reactive: Testing a Mediated-Moderation Model of Dark Triad and Perceived Victimization *via* Self-Regulatory Approach

**DOI:** 10.3389/fpsyg.2020.02141

**Published:** 2020-09-15

**Authors:** Hira Salah ud din Khan, Ma Zhiqiang, Shakira Huma Siddiqui, Muhammad Aamir Shafique Khan

**Affiliations:** ^1^School of Management, Jiangsu University, Zhenjiang, China; ^2^Adjunct Faculty Member Air University School of Management (AUSOM), Air University, Islamabad, Pakistan

**Keywords:** dark triad, perceived victimization, abusive supervision, mindfulness, mediated moderation model

## Abstract

Generally, it is difficult to work efficiently in a toxic environment. Surprisingly, leaders are found to be liable for such toxic atmosphere because they possess certain traits that employees perceive as victimization. This research assesses the relationship between the dark triad (narcissism, Machiavellianism, and psychopathy) and perceived victimization with a focus on the mediating effect of abusive supervision and the moderating effect of mindfulness. For this purpose, we surveyed 274 employees in the healthcare sector of Pakistan by using random sampling technique in three waves. To analyze the data, the structural equation model with partial least squares and PROCESS were used. The findings suggest that abusive supervision plays a mediating role in the association between the dark triad and perceived victimization. The results did not support the mediating role of abusive supervision in the association between narcissism and perceived victimization, however, the mediated moderation model was supported. Further, the findings suggest that mindfulness weakens the effect of abusive supervision on perceived victimization. Finally, the theoretical and practical implications of the results are also discussed.

## Introduction

There is divided opinion regarding whether victims invite abuse through their personality or behavior. As scholars consider this question, weighing its significance, it is argued that the instigator, and not the victim, is to be blamed for the abuse (Cortina et al., 2018). Accordingly, the literature concedes the instigators as toxic individuals who suck out positive energy and are non-supportive in the progress of an entity or the individuals in an organization (Templer, 2018). Paulhus and Williams (2002) defined “toxic employees” as those who score high on the dark triad (DT) traits; that is, employees with the underlying personality traits of Machiavellianism, narcissism, and psychopathy. Interestingly, possessing such traits does not seem to hinder individuals from achieving organizational power. On the contrary, some have claimed that such traits may help people engage in productive professions and get promotions to higher positions of power (Wisse and Sleebos, 2016). Yet, putting individuals who score high on the DT traits in managerial positions can possibly lead to substantial disaster (Wisse and Sleebos, 2016). In line with this, scholars have expounded that leaders with DT traits affect employees’ productivity and efficiency, leading to greater turnover and reduced performance (Aquino and Thau, 2009; O’Boyle et al., 2012). Indeed, an organization must endeavor to cognize perceived victimization that affects any of its workforce. It is worth mentioning that the literature echoes and emphasizes the relevance of discerning the behavior or traits of instigators who victimize their colleagues or subordinates by resorting to abusive behavior (Cropanzano et al., 2017), however, this has received little attention among scholars of organizational literature (Jensen and Raver, 2018; Hurst et al., 2019). Previous studies on victimization have focused on examining individual- and scenario-based antecedents comprising adverse psychological and physiological consequences and suggesting prevention and coping mechanisms for perceived victimization (Aquino and Thau, 2009). van Geel et al. (2017) found that employees experience more perceived victimization, while Muris et al. (2017) reported that individuals with DT traits are commonly involved in manipulating and abusing others to target their victims on the basis of their sharp propensity to understand the personality and psychological features of others (Lomas et al., 2019). Meta-analytic studies have linked the DT traits to adverse consequences, such as reduced well-being, efficiency, and engagement (O’Boyle et al., 2012; Muris et al., 2017).

In a quest to expand this knowledge, the present study draws insights from the social exchange theory by advancing the need to comprehend the factors that link the leader’s dark traits and the employee perceptions of the victimization phenomenon. Thus, this study aimed to contribute substantially to the understanding of the perceived victimization of employees by leaders with dark traits. To this end, this study first examines the key behavioral aspects of DT that serve as the basis of perceived victimization and instigate unfavorable work outcomes, which have been overlooked by previous studies, by examining the relationship between DT and perceptions of victimization. Though some scholars (van Geel et al., 2017) identified the link between DT and bullying, little has been said about its impact on perceived victimization (Lomas et al., 2019). The high prevalence of employees’ perceived victimization in modern workplaces instilled the need to explore the relationship between leaders’ dark traits and employees’ perceived victimization. Thus, we examine leaders’ DT traits and perceived victimization, which is an important missing link that affects employee outcomes (Baloch et al., 2017). In addition, Lomas et al. (2019) have shown that there are inconsistencies between the DT traits and their outome variables, including workplace bullying and perceived victimization, which highlights the need to build a firm relationship between these variables and to explore such relationship. Perceived victimization alludes to an individual’s acknowledgement or self-labeling and identifying oneself as a victim (Gupta and Bakhshi, 2018). Studies have indicated that perceived victimization (PV) is a contextual and perceptual mechanism (Aquino, 2000). For instance, employees receiving insufficient feedback from their bosses may believe that their leader is intentionally avoiding them (An et al., 2016). In line with this, scholars have characterized PV as the perception of being imperiled or maltreated (Jockin et al., 2001). This means that employees can encounter stress at work and perceive such stressors as deliberate and violent as opposed to being accidental and beyond the control of the perpetrator (An et al., 2016). Contrarily, researchers note that “bullying seems to exist on a continuum from occasional exposure to negative behaviors to severe victimization resulting from frequent and long-lasting exposure to negative behaviors at work” (Einarsen et al., 2010, p. 12). Moreover, scholars concede that bullying in the workplace is a significant predictor of a stressful atmosphere that leads to disastrous consequences by a developing perception of victimization in employees (Giorgi et al., 2016; Gupta and Bakhshi, 2018). Thus, PV is one of the possible outcomes of stressors that effect the employees (Gupta and Bakhshi, 2018). Therefore, this research further investigates the mediating factors that influence the relationship between DT and perceived victimization. Additionally, Tepper et al. (2017) argue that abusive supervision is prevalent in organizations and is partly instigated by workplace practices that cultivate feelings of envy.

Thus, abusive supervision represents the idea of victim’s perception. The theory of victim’s perception contends that certain people could be at risk of victimization by provoking (often unconsciously) the aggression of possible perpetrators (Tepper et al., 2006). Particularly, abusive supervisors exhibiting negative behaviors can potentially harm employees who are heavily reliant on them (Khan and Siddiqui, 2019). This empowers supervisors, giving them control over others’ actions (Kim et al., 2017). Such undesirable characteristic of DT may lead to perceived victimization because of the supervisor’s hostile attitude toward coworkers or subordinates (Baughman et al., 2012). Based on this argument, the present study examines the mediating role of abusive supervision on the DT-perceived victimization relationship.

The insufficient research and indecisive findings on the relationship between DT and perceived victimization indicate toward some individual characteristics and contextual factors which affect workplace behavior (Dadaboyev et al., 2019). However, nothing has been said about the contingencies that affect the relation between leaders’ DT and employees’ perceived victimization. Referring to the known facts about the important role mindfulness plays in organizations, this study investigates the moderating role of employee mindfulness in the relationship between abusive supervision and perceived victimization by looking through the lens of social exchange theory, which also endorsed self-regulatory mechanisms to demonstrate the reasons behind non-reciprocation to hostility due to personal differences (Tepper et al., 2017). Scholars have demarcated mindfulness as a psychological element that refers to the “awareness and observation of the present moment without reactivity or judgment” (Glomb et al., 2011, p. 116). The growing body of literature on mindfulness indicates that its core purpose is to enhance self-regulation of feelings, thoughts, and actions (Hülsheger et al., 2015; Aránega et al., 2019). As stated by Glomb et al. (2011), there are two basic elements of mindfulness: first, disentangle oneself from experience and, second, decrease sensitivity and automaticity. Basically, persons with a high degree of mindfulness may regulate their emotions by disassociating themselves from situations and avoiding reactive responses to incidents (VanVactor, 2012). This can be well understood by Thích Nhất Hȧnh’s famous quote: “The feeling that any task is a nuisance will soon disappear if it is done in mindfulness.” Researchers indicated that mindfulness helps an employee decenter oneself from stressful events and reevaluate the experiences cognitively in more positive ways (Garland et al., 2015). Studies have revealed that mindfulness significantly impacts the self-regulatory processes (Burton and Barber, 2019). Moreover, scholars highlighted the essential role of mindfulness as a significant psychological intervention that strengthens one’s emotion regulation mechanisms (Gunst et al., 2019). Self-regulation mechanism emphasizes the self-awareness aspect of self-regulation or perceptual processes (Vago and David, 2012). Numerous studies have demonstrated that mindfulness reduces the negative effects caused by stressors, such as abusive supervision, burnout, and retaliation, and uplifts the behavioral and psychological self-regulation of employees (Brown and Ryan, 2003; Roche et al., 2014; Long and Christian, 2015). Given such views, employees perceive low abusive supervision provided that mindfulness helps activate their self-regulatory mechanism during the occurrence of stressful events (Shapiro et al., 2006; Sutcliffe et al., 2016). Moreover, it is established that mindfulness helps in activating self-regulation, such as homeostasis, when confronted with physical or psychological stress by safeguarding the surrounding processes from the adverse effects of the stressor (Vago and David, 2012), which could be referred to as the “skiing effect” and as a supportive mechanism to self-regulatory processes; we attribute to it as “Big Brother.” The skiing effect acts as a metaphor to developing emotional regulation, or employees’ ability to understand their emotions as a resource to navigate life’s difficult moments; the Big Brother acts as a metaphor for support as it boosts the self-regulatory mechanism and helps one face any stressful environment. Based on such arguments, this study anticipates mindfulness to be a strong psychological intervention that helps stimulate the self-regulatory processes which are specifically essential for the assessment of social exchange relationships (unjust treatment appraisals) resulting to abusive supervision and perceived victimization. Thus, we contend that mindfulness, by virtue of its self-regulatory perspective, can alleviate the harmful effect of abusive supervision on the dark traits of leaders, which in turn enhances organizational success and leads to reduced feelings of victimization.

**FIGURE 1:**
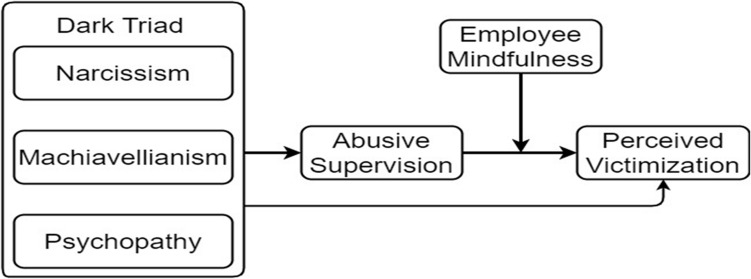
Conceptual Framework.

Considering the above, this study contributes to the existing body of knowledge on DT and perceived victimization by framing a conceptual model that encapsulates abusive supervision as a mediating mechanism of the relationship between DT and perceived victimization by using the social exchange theory. Furthermore, this study investigates the moderating effect of employee mindfulness as well as the mediated moderation model in the healthcare sector in Pakistan, as can be seen in Figure 1.

## Literature Review

### Dark Triad

The concept of DT was first highlighted by Paulhus and Williams (2002) who studied the dark personality traits and discussed its prominent features, which are characteristically distinct. Researchers explained that DT refers to the personality traits of narcissism, psychopathy, and Machiavellianism, which reflect malicious aspects of personality (Cohen, 2016). Narcissism is characterized by self-importance, self-love, flattery, and fantasies of controlling others to gain success and admiration. Machiavellianism is characterized by clever manipulation of others and placing oneself before moral standards/principles (Stiff, 2019). Studies indicated psychopathy as the worst of all the DT traits that is characterized by lacking feelings of regret or guilt in sabotaging others and having no empathy or concern for anyone (O’Boyle et al., 2012). Thus, regarding moral values, DT traits have been marked as a significant factor leading to hostility and neglect (Muris et al., 2017). For instance, studies have revealed that narcissism is positively linked to many negative work outcomes, such as provoked aggression and counterproductive work behaviors. Both Machiavellianism and psychopathy are found to be positively correlated with counterproductive work behaviors and negatively with job performance (Cohen, 2016). However, further investigation on how DT is linked with leaders and what are its consequences will enrich the literature on DT and will add to our understanding of differences in personality traits.

### Relationship Between DT and Perceived Victimization

Literature review reveals that DT traits are linked with essential organizational outcomes, such as career success and the overall well-being of employees (O’Boyle et al., 2012; Cohen, 2016). Schyns and Schilling (2013) found that workers fall victims to bad leadership because of inadequate resources to protect themselves. Leaders with DT traits do not have empathy; therefore, they make biased decisions contrary to the needs and requirements of their employees (Muris et al., 2017). Scholars identified that in the long term, narcissists are assessed as negative-minded people and are left off to suffer the consequences (Kwiatkowska et al., 2019). In line with this, scholars have shown that narcissistic leaders have bad relationships with their subordinates, which adversely effects job satisfaction and other work outcomes (Shurden and Shurden, 2019). On the other hand, psychopaths feel detached from the environment and seek self-enrichment (Blickle and Schütte, 2017). As stated by Kowalski et al. (2018), leaders with psychopathic tendencies do not hesitate manipulating the employees to their advantage. Psychopathic individuals desire hurting others and facilitate unlawful and other rebellious behaviors (O’Boyle et al., 2012). Psychopathic leaders are, thus, likely to victimize their subordinates for multiple gains (Murphy et al., 2017). Machiavellians are exploiters who selfishly manipulate others to gain their objectives (Stiff, 2019). Scholars pair Machiavellianism with a high degree of control and callousness (Kiazad et al., 2010). Given this theory, DT and social exchange approaches employ an analogous logic beneficial for understanding workforce responses toward leaders with DT traits. Scholars further revealed that strain purports displaying a negative assessment of worker–organization exchange association (Cropanzano et al., 2017). In conformity with the above-mentioned arguments, we assume that leaders’ narcissism, psychopathy, and Machiavellianism traits are positively related to perceived victimization in the workplace. Thus, we postulate the following:

H1a: *Narcissism is significantly and positively related to perceived victimization.*

H1b: *Machiavellianism is significantly and positively related to perceived victimization.*

H1c: *Psychopathy is significantly and positively related to perceived victimization.*

### Mediating Role of Abusive Supervision

Abusive supervision refers to “subordinates’ perceptions of the extent to which their supervisors engage in the sustained display of hostile verbal and nonverbal behaviors, excluding physical contact” (Tepper, 2000, p. 178). Abusive supervision is a form of destructive leadership (Khan and Siddiqui, 2019). Veteran scholars stated that abusive supervision is perceived as unfair and has adverse impact on subordinates’ outcomes (Tepper et al., 2017). According to the social exchange theory, this happens due to transgressions of social exchange relationships that result in perceptions of unfairness, which are derived from personal gains (Cropanzano et al., 2017). The leader–subordinate relationship depends on a social exchange relationship that brings harmony and mutual gains concerning fair dealings (Kudesia and Reina, 2019). According to Wisse and Sleebos (2016), dark personality traits lead to social exchange violation as employees respond to supervisor abuse that includes unfair and insulting treatment. Moreover, employees reciprocate by getting even with the supervisor. Thus, abusive supervision is socially and psychologically unacceptable to employees (Paulhus and Williams, 2002). Literature on leadership has documented that some leaders have destructive behavior (Schyns and Schilling, 2013). There are leaders with an inborn desire to use an abusive style of leadership (Breevaart and de Vries, 2017). Each DT trait has its distinct features, which have diverse impacts on work outcomes. For example, narcissism is described as reactionary; when narcissists receive undesirable feedback, their self-esteem is hurt (Casale et al., 2019). Corroborating this, researchers have verified that unfavorable feedback and confrontation force supervisors to practice abusive supervision (Long and Christian, 2015). Machiavellianism, another DT trait, is a cluster of specific features such as cynicism, brutality, lack of moral beliefs, argentic goals, manipulation, and exploitation (Stiff, 2019). In view of these features and the social exchange theory, it is considered that Machiavellian leaders tend to use abusive supervision to achieve vested interests (Kiazad et al., 2010). Lastly, psychopaths are people who enjoy and practice thrill, are insensitive, show lack of remorse, and display cynical behaviors in general (Murphy et al., 2017). In view of this, psychopathic leaders are those who use abusive supervision to gain authority by sabotaging others’ rights (Kiazad et al., 2010; Murphy et al., 2017). Growing literature on DT has shown that leaders having one of these traits are futile to the organization (Jonason et al., 2012). Scholars further found that DT is negatively linked to empathy, thus allowing supervisors to abuse the workforce while showing no empathy for the victims (Jones and Paulhus, 2014). Organizations should pay heed to the perceptions of victimization of employees because of abusive supervision as it effects their well-being and productivity (Aquino and Thau, 2009). Although research has revealed that employees are victimized by certain toxic personalities, most of the studies overlooked the mechanisms or the factors that lead people with such dark personalities to victimize employees in an organization (Cohen, 2016). Considering this, we argue that abusive supervision might be a significant intervening construct in the relationship between DT and perceived victimization. Some scholars have established that a social exchange cycle initiates when an organizational focal person or offender, generally a superior or a colleague, interacts with the target individual, either positively or negatively (Cropanzano et al., 2017). Research indicates that the workforce reduces putting in effort and time to work in response to the imbalance in the exchange relationship and that employees are most likely to avoid the situation which is perceived as harmful or unfavorable (Kim et al., 2017). Thus, we postulate the following:

H2a: *Abusive supervision mediates the relationship between narcissism and perceived victimization*.

H2b: *Abusive supervision mediates the relationship between Machiavellianism and perceived victimization*.

H2c: *Abusive supervision mediates the relationship between psychopathy and perceived victimization*.

### Employee Mindfulness as a Moderator in the Relationship Between Abusive Supervision and Perceived Victimization

Abusive supervision manifests in destructive social exchanges between bosses and subordinates, leading to malicious response from the workforce as a result of maltreatment (Tepper et al., 2017). Yet, subordinates’ behavior is not always rebellious toward the negative attitude of the supervisors. Researchers have endorsed self-regulatory mechanisms into the social exchange theory to demonstrate the reasons behind this non-reciprocation to hostility (Long and Christian, 2015). Although studies have highlighted the negative impact of abusive supervision on employee outcomes (Khan and Siddiqui, 2019), few have focused on personal differences in self-regulation behaviors (Tepper et al., 2017). Researchers consider mindfulness to be a positive intervention for uplifting the self-regulatory mechanisms pertaining to abusive supervision since mindfulness successfully helps mitigate adverse effects (Hafenbrack, 2017). Moreover, mindfulness has been acknowledged to diminish negative mental and psychological responses when encountered with injustice in the workplace (Long and Christian, 2015).

Scholars concede that mindfulness is a psychological term linked to cognizance and alertness of the current event without being reactionary or judgmental (Hafenbrack, 2017). This concept originated from Buddhism (Marques, 2012) and has recently gained much popularity in the academic arena (Lundwall et al., 2019). Contemporary studies have laid much emphasis on the importance of mindfulness in the workplace (Hafenbrack, 2017). The most prominent aspect of mindfulness is that “mindfulness and mindfulness-based practices lead to improved self-regulation, and ultimately, higher functioning” (Glomb et al., 2011, p. 124). Glomb et al. (2011) elaborated that persons with higher mindfulness can bring “a decoupling of the self (i.e., ego) from events, experiences, thoughts, and emotions” (p. 124). Moreover, such persons encounter “a decrease in automaticity of mental processes in which past experiences, schemas, and cognitive habits constrain thinking” (p. 124), which makes them less perceptive to details and diminishes the impulsive response to unfavorable situations (Aránega et al., 2019). Thus, mindfulness is the ability to respond smartly to a hostile work environment by a strong self-regulatory mechanism (Lundwall et al., 2019). Consequently, based on the self-regulatory approach, we propose that mindfulness can buffer the harmful effects of abusive supervision on perceived victimization at work. As mentioned earlier, abusive bosses who generally criticize or scorn their subordinates mar the confidence of employees and victimize them inadvertently. Mindfulness can help employees disconnect themselves from hostility. Instead of viewing the negative impact of abusive supervision regarding self, employees having high mindfulness can objectively look at the events. On the contrary, employees having lesser mindfulness tend to perceive themselves as victims on account of the abusive supervision at work, and thus their confidence is shattered. Researchers found that mindfulness decreases the impact of decisions involving discrimination (Long and Christian, 2015). Many studies have focused on the significance of mindfulness in employees that helps them improve their ability to meet challenges in the competitive world (Hülsheger et al., 2014; Zheng and Liu, 2017). Literature review indicates that mindfulness helps one cope with psychological stressors that cause depression and other emotional impairments (Ndubisi, 2012; Lundwall et al., 2019). Corroborating this with the social exchange theory, mindfulness helps the workforce dispense positive social interactions, facilitates performance, promotes workers’ well-being, and plays a vital role in the leader–follower association (Coo and Salanova, 2018; Kudesia and Reina, 2019). Considering the previously mentioned arguments, we postulate that:

H3: *Mindfulness moderates the relationship between abusive supervision and perceived victimization at work, such that the relationship is weaker for those with high rather than low degree of mindfulness*.

### Mediated Moderation Model

Infusing a self-regulatory perspective into the social exchange theory, our investigation offers an integrated mediated moderation model in which abusive supervision mediates the moderating role of mindfulness in the relationship between DT and perceived victimization. Though few studies have emphasized the intervention approaches that help workers efficiently cope with abusive supervisors (Tepper et al., 2017), devising strategies to overcome the adverse impact of abusive supervision is essential to control personal and monetary costs (Tepper et al., 2006). Therefore, we expand the literature on DT by investigating the interacting role of mindfulness. Self-regulatory mechanism in mindful subordinates plays an important role in reducing the impact of stressful events, which results in decreased perceptions of victimization (Lomas et al., 2019). On the contrary, subordinates with a low degree of mindfulness are prone to be targeted by abusive supervision, which results in increased perceptions of victimization. To sum up the argument, we posit that:

H4a: *Mindfulness moderates the mediated relationship between narcissism and perceived victimization through abusive supervision in such a way that a higher level of mindfulness will weaken the indirect relationship*.

H4b: *Mindfulness moderates the mediated relationship between Machiavellianism and perceived victimization through abusive supervision in such a way that a higher level of mindfulness will weaken the indirect relationship*.

H4c: *Mindfulness moderates the mediated relationship between psychopathy and perceived victimization through abusive supervision in such a way that a higher level of mindfulness will weaken the indirect relationship*.

## Materials and Methods

### Sample and Procedures

The data was obtained from nurses working in hospitals in Pakistan. We chose hospitals to conduct the study because healthcare staff needs a conducive environment to look after the patients. If the nurses’ own mental and psychological well-being is threatened by abusive supervision, they would not be able to perform their duties well. A questionnaire survey technique was adopted to collect the data. Referring to earlier studies, English was chosen as the appropriate medium for the survey procedure (Khan et al., 2016). Once permission was obtained from the target hospitals, we contacted all the staff on duty one by one, inviting them to participate in the investigation. We then visited each of the target hospitals and directed a paper-based questionnaire to potential participants. The survey included a cover letter explaining the purpose of the research and the concept of voluntary participation, assuring participants of privacy.

The study received the support of the entire higher management and assistance was provided by the human resource departments of the respective hospitals. To deal with the possibility of common method biases (Podsakoff et al., 2003, 2012), we gathered data at three time points (i.e., time 1, time 2, and time 3), with a lag of 2 months in each wave of data collection. This is an example of “an incorporate (i.e., bigger than two or three) wave of data with relatively short time lags” (Frone et al., 1997, p. 330). Studies have indicated that collecting data in three waves helps the search and discovers the causal effect of variables (Frone et al., 1997), while Peng (2013) suggested that a 2-month lag is sufficient to reduce the common method bias. In the present study, in wave 1 of data gathering, questionnaires were distributed to 734 nurses, selected randomly from the target hospitals. The questionnaires aimed to gather data concerning respondents’ DT traits and demographics (e.g., age, gender, and tenure). The data concerning the respondents’ DT traits were gathered as a proxy to leaders of employees working in different departments of the hospitals. The subordinates filled in the information regarding abusive supervision, mindfulness, and perceived victimization. To ensure confidentiality, the respondents were asked to enter fake names or codes on the questionnaires. For all the three types of questionnaires, the respondents were asked to provide the same code or nickname. In total, we obtained 621 responses. A couple of months later, we executed the second wave (T2) of data collection. Questionnaires concerning abusive supervision and employee mindfulness were again administered to the same 734 nurses who responded during the first wave. We obtained a total of 608 responses in T2. The third wave was conducted 2 months after T2. A survey concerning perceived victimization was sent to the same 734 participants, out of which 535 responded. The code and code names of each respondent were checked by the researchers, matching them to the questionnaires that bore the same code name. We also checked the demographics to identify and match the questionnaires filled out by the same participants in each wave. Thus, we were left with 279 questionnaires that matched in all the three waves. Out of these, five questionnaires, which were not properly filled out, were dropped; finally, we were left with 274 valid samples.

A total of 65 leaders completed the surveys which comprise the following demographic information: 19% of them are males while 4.7% are females. Their age ranges are as follows: 0.2% were 20–30 years, 8.5% were 31–40 years, 11.2% were 41–50, and 3.7% were over 50 years. The leader’s job tenure was the same as that of the subordinates: 1–5 years.

A total of 209 focal subordinates, who were working under immediate leaders, represented the following demographic details: male, 54%; females, 22%. Of the subordinates, 2.9% were below 20 years, 19.7% comprised 20–30 years, 39.1% were 31–40 years, 9.1% were 41–50 years, and 5.5% were over 50 years. The subordinates’ job tenure details are as follows: 17.9% of them had less than 1 year, 48.2% had 1–5 years, and 10.2% had over 5 years.

### Measures

The data were collected using a self-administered questionnaire. A self-report study is a type of survey, questionnaire, or poll in which the respondents read the question and select a response by themselves without interference (Jupp, 2006). A five-point Likert scale ranging from “1 = strongly disagree to 5 = strongly agree” was used to rate the responses. The present study views perceived victimization in terms of subjectively perceiving oneself as a victim of dark triad leaders at work. Burgeoning studies have stated that perceived victimization jeopardizes the resources that employees link with themselves, like psychological contentment or well-being, which has been related to stress (Lewis and Orford, 2005; Bowling and Beehr, 2006; Aquino and Thau, 2009; Bentley et al., 2017). Scholars noted that PV directly affects the performance as the latter is often rooted within the societal perceptions and resources in the relational atmosphere of the workplace, particularly when perceived in distinctive dyadic interfaces (Bentley et al., 2017). In view of this, employees’ ability to control and regulate their interpersonal resources is crucial in shaping their attitudes (Khan et al., 2019) and behavior when they perceive victimization (Bentley et al., 2017). Therefore, in this study, we focused on perceptions of subordinates of being victimized by their leaders. Temporary segregation of responses by using the time lag method enabled reducing the common method biases and improved our confidence in causality predictions (Podsakoff et al., 2012; Miller et al., 2017). Scholars have demonstrated that a reliability test is vital when assessing the goodness of the collected data. Cronbach’s alpha is defined as a reliability coefficient that demonstrates the positive relationship of one component to another in an array (Sekaran, 2000). It is explained that the instrument has a higher reliability when the estimate of Cronbach’s alpha is 1.000 (Sekaran, 2000). Furthermore, Sekaran (2003) attested that the estimate of reliability with a Cronbach’s alpha lower than 0.60 is marked as poor, whereas estimates in the range of 0.70 are considered as acceptable and above 0.80 indicates good. In present study, validity and reliability scales were used based on previous studies. Given this, prior studies have reported adequate estimates of reliability for each subscale of the DT measure, which indicated Cronbach’s alpha values greater than 0.70 for Machiavellianism, narcissism, and psychopathy (Kiazad et al., 2010; Jones and Paulhus, 2014; Baloch et al., 2017; Turner et al., 2019). In a similar way, previous studies have demonstrated adequate Cronbach’s alpha values exceeding 0.70 for the abusive supervision scale (Haggard and Park, 2018; Low et al., 2019; Shillamkwese et al., 2019). Similarly, previous studies have validated the reliability of the scales used to assess the perceived victimization scale (Parker, 2019; Tarraf et al., 2019). Moreover, extensive studies have reported the reliability of the use of the employee’s mindfulness construct (Baron, 2016; Zheng and Liu, 2017; Anasori et al., 2019). The scales adopted for the current study were used in multidisciplinary studies that focused on behavioral perspectives.

### Dark Triad

Leaders’ DT traits were measured using the Short Dark Triad (SD3) Scale (Jones and Paulhus, 2014). To this end, a questionnaire was administered to gather data from employees about their leaders’ dark traits. The questionnaire comprised 27 items, with nine items for each DT trait. For instance, narcissism-related items included: “My supervisor likes to get acquainted with important people” and “My supervisor insists on getting the respect he/she deserves.” Machiavellianism-related items included: “My supervisor likes to use clever manipulation to get his/her way.” Sample items for the psychopathy trait included: “My supervisor likes to get revenge on authorities” and “It is true that he can be mean to others.” Previous studies have reported adequate coefficient alpha values for each of these SD3 subscales: 0.71 for narcissism, 0.77 for Machiavellianism, and 0.80 for psychopathy (Jones and Paulhus, 2014). The present study found that the SD3 had adequate coefficient alphas for each of the subscales: 0.86 for narcissism, 0.87 for Machiavellianism, and 0.88 for psychopathy.

### Perceived Victimization

Victimization of employees in the workplace was measured using an eight-item scale of perceived victimization (Sasso, 2013). The respondents were asked to remember an event at their place of employment in which they witnessed violence or conflict. They were then provided with eight items intended to express emotions both during and after the unpleasant event. The sample items for this measure included: “I was intentionally treated poorly” and “I felt deliberately accosted.” Previous studies have reported Cronbach’s alpha of 0.94 to represent the adequate reliability of this scale (Sasso, 2013). The current study showed a Cronbach’s alpha of 0.92.

### Abusive Supervision

Employees scored their leaders using a 15-item Abusive Supervision Scale (Tepper, 2000). Sample items included: “My boss ridicules me” and “My boss tells me that my thoughts or feelings are stupid.” The Cronbach’s alpha reliability indicated by previous studies was 0.90 (Breevaart and de Vries, 2017). The present study also demonstrated a sufficient alpha reliability of 0.92.

### Employee Mindfulness

This construct was assessed using a 15-item Mindful Attention Awareness Scale (Brown and Ryan, 2003). The sample items included: “I could be experiencing some emotion and not be conscious of it until sometime later” and “I break or spill things because of carelessness, not paying attention, or thinking of something else.” Previous studies have reported the reliability of this measure to be adequate at 0.80 (Fredrickson et al., 2008; Zheng and Liu, 2017). The Cronbach’s coefficient for the present study was found to be 0.93.

### Control Variables

Numerous studies on perceived victimization have indicated that certain workforce demographic variables, such as gender, age, and tenure, have a tendency to impact the perceived victimization of employees (Aquino et al., 1999; Aquino and Bradfield, 2000; Bowling and Beehr, 2006; Parker, 2019). Therefore, we controlled for leaders’ and subordinates’ characteristics, for instance age, gender, and job tenure, in the present study.

## Analysis Strategy

To test the hypotheses of this study, we employed both partial least squares structural equation modeling (PLS-SEM) and PROCESS macros. Scholars have demonstrated that the PLS technique is predicated on the structural equation model (SEM) and the measurement model (Henseler, 2017b). PLS is an appropriate data analysis technique for this study because of the measurement model and sample data characteristics. In this study, the measurement model has a small sample size (*n* = 274) and have few indicators (<6), which are suitable for the PLS algorithm (Hair et al., 2017). The research model entails considerable complexity concerning the types of relationships in the hypotheses. The measurement model used in the current study are composites based on scales developed by scholars (Henseler, 2017a). Moreover, this research not only predicts but also elaborates the differences among the target measures. The application of PROCESS macro developed by Hayes (2013) is more suitable for a mediated moderation analysis. Therefore, we believe that they can be suitable analysis techniques for the current research (Carrion et al., 2016; Felipe et al., 2016; Baloch et al., 2017; Naguib and Naem, 2018).

### The Measurement Model

SmartPLS was used to evaluate the measurement model; item loading, rho-A, average variance extracted (AVE), variance inflation factor (VIF), and discriminant validity were measured. The results showed that AS7, AS12, EM9, EM12, EM13, and PV5 were removed from the final analysis of the dataset because of weak loading values. Table 1 shows that the factor loadings of all the indicators are greater than the threshold value of 0.7, thereby confirming the reliability of the measurement model. As structural testing is imperative for the reliability and validity of the measurement model (Henseler et al., 2009), we calculated the Dijkstra–Henseler’s rho indicators to test the construct validity (Dijkstra and Henseler, 2015). The results show that the reliability values of all the composite indicators are greater than the threshold value of 0.7 as suggested by Henseler (2017a). Furthermore, convergent and discriminant validities of the latent variables were also found satisfactory. Multicollinearity was also tested through VIF values. All the values were found to be less than 5, thereby confirming that multicollinearity was not an issue in the model (Kim, 2019). Table 2 shows the square root values of AVE on the diagonal (in bold), which confirm the discriminant validity according to the widely used criterion of Fornell–Larcker. Moreover, according to the heterotrait–monotrait ratio (HTMT), the values of all the constructs were under the threshold point of 0.85. Therefore, we can say that the obtained values provide evidence of discriminant validity.

**TABLE 1:** Measurement model.

**Construct/dimension/indicator**	**VIF**	**Loadings**	**Rho_A**	**AVE**
Narcissism	1.494		0.888	0.709
N1		0.856		
N2		0.885		
N3		0.896		
N4		0.718		
Machiavellianism	1.616		0.914	0.725
M1		0.877		
M2		0.896		
M3		0.834		
M4		0.795		
Psychopathy	1.427		0.952	0.739
P1		0.922		
P2		0.899		
P3		0.870		
P4		0.736		
Abusive supervision	1.406		0.870	0.509
AS1		0.803		
AS2		0.814		
AS3		0.738		
AS4		0.752		
AS5		0.749		
AS6		0.772		
AS7		0.122^*a*^		
AS8		0.717		
AS9		0.740		
AS10		0.822		
AS11		0.737		
AS12		0.472^*a*^		
AS13		0.718		
Employee mindfulness	1.792		0.943	0.533
EM1		0.789		
EM2		0.789		
EM3		0.765		
EM4		0.782		
EM5		0.757		
EM6		0.795		
EM7		0.804		
EM8		0.740		
EM9		0.206^*a*^		
EM10		0.746		
EM11		0.780		
EM12		0.691^*a*^		
EM13		0.671^*a*^		
EM14		0.704		
EM15		0.722		
Perceived victimization			0.946	0.680
PV1		0.871		
PV2		0.875		
PV3		0.819		
PV4		0.899		
PV5		0.506^*a*^		
PV6		0.879		
PV7		0.884		
PV8		0.790		

*All loadings are significant at the 0.001 level (two-tailed). rho_A, Dijkstra–Henseler’s rho indictors; VIF, variance inflation factor; AVE, average variance extracted. ^*a*^The items were removed from the final version of the construct and not used in the structural model.*

**TABLE 2:** Measurement model: discriminant validity.

**S. no.**		**Fornell–Larcker criterion**						**Heterotrait–monotrait ratio (HTMT)**					
		**1**	**2**	**3**	**4**	**5**	**6**	**1**	**2**	**3**	**4**	**5**	**6**
1	Narcissism	**0.842**											
2	Machiavellianism	-0.130	**0.851**					0.172					
3	Psychopathy	0.436	0.045	**0.860**				0.492	0.072				
4	Abusive supervision	-0.112	0.519	0.095	**0.713**			0.147	0.553	0.122			
5	Employee mindfulness	0.416	0.376	0.471	0.265	**0.730**		0.458	0.405	0.254	0.493		
6	Perceived victimization	0.438	0.238	0.282	0.105	0.662	**0.824**	0.475	0.245	0.122	0.698	0.280	

*Values in bold are the square roots of the average variance extracted (AVE).*

### The Structural Model

Table 3 shows the impact of one variable on another variable measured by using the estimated values of path coefficients. In the current analysis, we used bootstrapping technique by randomly drawing 5,000 subsamples at the significance level of 0.05%. Bootstrapping measures the statistical significance of the relationship between variables by providing confidence intervals and producing standard errors (Hair et al., 2016). The structural equation modeling analysis was completed in four steps by using four models. In model 1, we determined the total effect of independent variables (narcissism, Machiavellianism, and psychopathy) on the dependent variable (perceived victimization). In model 2, abusive supervision was introduced as a mediator between the independent and dependent variables. In model 3, mindfulness was added as a moderator variable. In model 4, the interaction between the mediator and the moderator was assessed to analyze the mediation–moderation effect on the study variables.

**TABLE 3:** Structural model paths.

**Relationships**	**Model 1**	**Model 2**	**Model 3**	**Model 4**	***F*^2^**	**Support**
			
		*R*^2^_*A*__*S*_ = 0.65 *Q*^2^ = 0.58	*R*^2^_*A*__*S*_ = 0.36 *Q*^2^ = 0.10	*R*^2^_*A*__*S*_ = 036 *Q*^2^ = 0.10		
				
	*R*^2^_*P*__*V*_ = 0.39 *Q*^2^ = 0.36	*R*^2^_*P*__*V*_ = 0.40 *Q*^2^ = 0.38	*R*^2^_*P*__*V*_ = 0.35 *Q*^2^ = 0.32	*R*^2^_*P*__*V*_ = 0.38 *Q*^2^ = 0.34		
H1a: N → PV	0.274** (3.23) [0.12–0.43]	0.287** (3.17) [0.12; 0.47]	0.287** (3.17) [0.12–0.47]	0.287** (3.17) [0.12–0.47]		Yes
H1b: M → PV	0.228*** (3.95) [0.12–0.34]	0.116* (2.04) [0.06–0.22]	0.116* (2.04) [0.06–0.22]	0.116* (2.04) [0.06–0.22]		Yes
H1c: P → PV	0.346*** (5.34) [0.22–0.48]	0.229*** (3.46) [0.09–0.35]	0.229*** (3.46) [0.09–0.35]	0.229*** (3.46) [0.09–0.35]		Yes
N → AS		−0.057^*ns*^ (1.23) [−0.14 to.05]	−0.057^*ns*^ (1.23) [−0.14 to 0.05]	−0.057^*ns*^ (1.23) [−0.14 to 0.05]		
M AS		0.492*** (13.12) [0.42-0.56]	0.492*** (13.12) [0.42–0.56]	0.492***(13.12) [0.42–0.56]		
P → AS		0.513*** (13.47) [0.43–0.58]	0.513*** (13.47) [0.43–0.58]	0.513***(13.47) [0.43–0.58]		
AS → PV		0.228** (2.49) [0.04–0.42]	0.414** (6.51) [0.28–0.53]	0.461** (8.06) [.34–0.56]		
EM PV			0.302*** (5.31) [0.19–0.40]	0.267*** (4.19) [0.14–0.38]		
H3:ASx EM → PV				−0.159*** (3.19) [−0.25 to −0.06]	0.04	Yes

t-values in parentheses. Bootstrapping bias-corrected 95% confidence intervals are in square brackets (based on n = 5,000 subsamples). N, narcissism; M, Machiavellianism; P, psychopathy; AS, abusive supervision; EM, employee mindfulness; PV, perceived victimization; ns, not significant. *p < 0.05, **p < 0.01, *** < 0.001 [based on t(4999), two-tailed test].

**FIGURE 2:**
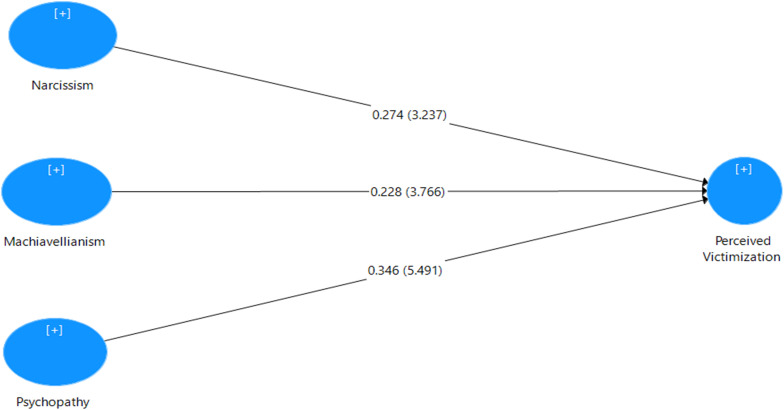
Structural model for direct relationship.

**FIGURE 3:**
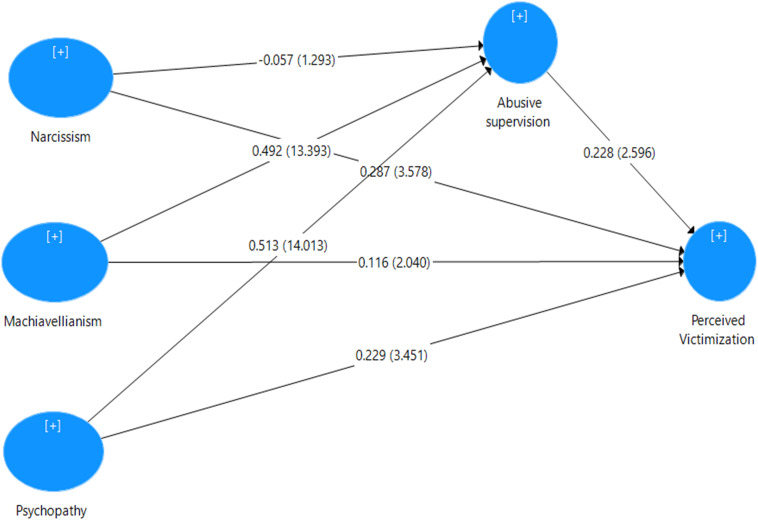
Structural model for Mediation paths.

**FIGURE 4:**
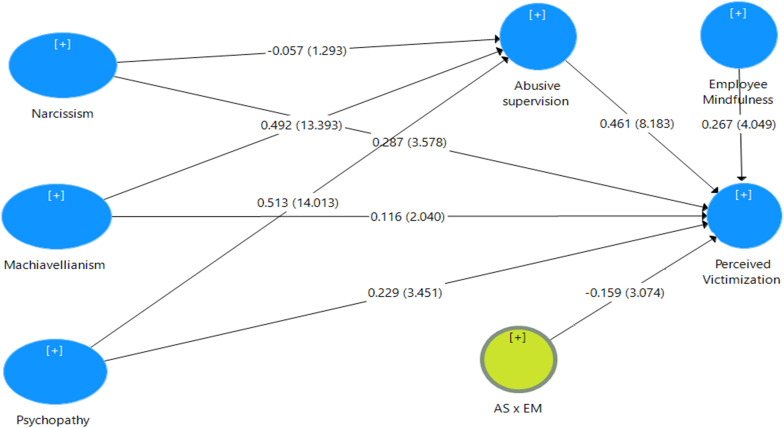
Structural model for Moderation paths.

The results of model 1 show that narcissism, Machiavellianism, and psychopathy have significant positive impacts on perceived victimization, as shown in Figure 2. The study hypotheses H1a, H1b, and H1c are thus statistically accepted. Furthermore, after including abusive supervision as a mediator in model 2, narcissism was found to have an insignificant impact on perceived victimization. However, Machiavellianism and psychopathy had a positive and significant direct and indirect impact, respectively, on perceived victimization. It was noted that the direct impact of Machiavellianism and psychopathy decreased after including the mediator in step 2. The study hypothesis H2a is thus statistically insignificant, while H2b and H2c are statistically accepted, as shown in Figure 3. Model 3 shows that mindfulness has a significant impact on perceived victimization. Model 4 shows that the interaction terms abusive supervision and mindfulness have a significant impact on perceived victimization. Importantly, a small *F*-square value cannot be neglected as it can predict a substantial effect. “If there is a likelihood of occurrence for the extreme moderating conditions and the resulting beta changes are meaningful, then it is important to take these situations into account” (Ali and Park, 2016). As 0.025, 0.01, and 0.005 are considered as large, medium, and small effect sizes, respectively, the effect size of H3 is large. Thus, hypothesis H3 is accepted, as shown in Figure 4.

After completing the analysis by using SmartPLS, we used Hayes Process 2017 to test the indirect effect of the independent variables on the dependent variable. To assess the significance level of the mediating effects, a bootstrapping procedure was used (Preacher and Hayes, 2008). We used a confidence interval of 95% to draw 5,000 subsamples. Table 4 shows that the total effect (0.384) of narcissism is significant, with a significant direct (0.292) but an insignificant indirect (0.092) effect on perceived victimization. Thus, H2a is insignificant because the direct impact of narcissism is greater than the indirect impact of abusive supervision on the perceived victimization. Furthermore, the total effect (0.524) of Machiavellianism is also significant, with a significant direct (0.231) and indirect (0.293) effect on perceived victimization. Thus, H2b is significant because the indirect impact of Machiavellianism is greater than the direct impact on the perceived victimization. In addition, it was found that abusive supervision partially mediates the relationship between Machiavellianism and perceived victimization. The total effect (0.420) of psychopathy is also significant, with a significant direct (0.199) and indirect (0.221) effect on perceived victimization. Thus, H2c is significant because the indirect impact of psychopathy is greater than the direct impact on the perceived victimization. In addition, it was found that abusive supervision partially mediates the relationship between psychopathy and perceived victimization.

**TABLE 4:** Summary of mediating effect tests.

**Path**	**Total effects on PV**				**Path**	**Direct effects on PV**				**Path**	**Indirect effects on PV**				**Support**
	**Effect**	***t***	**BCCI**			**Effect**	***t***	**BCCI**			**Effect**	***t***	**BCCI**		
			**Lower**	**Upper**				**Lower**	**Upper**				**Lower**	**Upper**	
N	0.384***	7.76	0.29	0.48	N	0.292***	6.54	0.20	0.38	N > AS > PV	0.092^*ns*^	1.24	0.03	0.18	No
M	0.524***	8.44	0.40	0.65	M	0.231**	3.01	0.08	0.38	M > AS > PV	0.293***	6.51	0.16	0.42	Yes
P	0.420***	8.91	0.33	0.51	P	0.199*	3.28	0.08	0.32	P > AS > PV	0.221***	3.03	0.14	0.30	Yes

t-values in parentheses. Bootstrapping bias-corrected 95% confidence intervals in square brackets (based on n = 5,000 subsamples). N, narcissism; M, Machiavellianism; P, psychopathy; AS, abusive supervision; PV, perceived victimization; BCCI, bias-corrected confidence interval; ns, not significant. *p < 0.05, **p < 0.01, ***p < 0.001 [based on t(4999), two-tailed test].

In the last step, PROCESS (Hayes, 2013) was utilized to assess the conditional effects of the independent variables on perceived victimization. The process generated estimates and 95% confidence interval bootstrap was performed to check the mediation–moderation effect on the dependent variable. Table 5 shows the indirect effect of the DT on PV *via* abusive supervision (AS) at values of the moderator (employee mindfulness) on the different variables. The results show that the value of the moderator positively increased, but the indirect effect decreased in all the relationships. Hence, the results support the study hypotheses H4a, H4b, and H4c.

**TABLE 5:** Conditional indirect effect analyses: conditional indirect effects of psychopathy, narcissism, and Machiavellianism on perceived victimization (PV) through abusive supervision (AS) at values of employee mindfulness as moderator.

**Mediator**	**Employee mindfulness**	**Indirect effect**	**Boot SE**	**BCCI**	
				**Lower**	**Upper**
AS (N)	3.733	0.105	0.043	0.023	0.187
AS (N)	4.400	0.076	0.031	0.022	0.143
AS (N)	4.867	0.056	0.030	0.012	0.130
AS (M)	3.733	0.412	0.063	0.289	0.537
AS (M)	4.400	0.257	0.057	0.145	0.366
AS (M)	4.867	0.149	0.078	-0.012	0.295
AS (P)	3.733	0.333	0.053	0.233	0.445
AS (P)	4.400	0.193	0.038	0.118	0.269
AS (P)	4.867	0.095	0.055	-0.014	0.198

*N, narcissism; M, Machiavellianism; P, psychopathy; BCCI, bias-corrected confidence interval.*

Table 6 shows the values of an index of mediated moderation for narcissism, Machiavellianism, and psychopathy. The results are also significant for all the variables as a zero value does not exist between both CI ends (Hayes, 2013).

**TABLE 6:** Conditional indirect effect analyses: index of mediated moderation.

**Mediator**	**Index**	**Boot SE**	**BCCI**	
			**Lower**	**Upper**
AS (N)	−0.044	0.031	−0.095	−0.035
AS (M)	−0.232	0.080	−0.390	−0.074
AS (P)	−0.210	0.069	−0.356	−0.080

*N, narcissism; M, Machiavellianism; P, psychopathy; BCCI, bias-corrected confidence interval.*

## Discussion

The purpose of this study was to examine the impact of the DT traits of leaders on perceived victimization and the mechanisms that affect the relationship between DT and perceived victimization. Our results demonstrate that narcissism is positively linked to perceived victimization, consistent with Salazar (2016). Thus, the results are consistent with the social exchange theory which holds that leaders that practice selfishness bring about antagonistic responses from subordinates. Further, our results show that Machiavellianism is positively linked to perceived victimization, indicating that individuals with this kind of personality trait lack moral values to collaborate with other employees. These results provide further support to previous studies and propose that leaders use their dirty cleaver to appeal to the emotions of subordinates in an organization in order to achieve their parochial interest, consistent with Stiff (2019). Moreover, the relationship between psychopathy and perceived victimization is also found to be highly significant, revealing that psychopathic personalities have aggressive behavioral tendencies. These results supported previous studies which indicated that the personality traits of offenders are usually similar to those with DT personalities (Jonason et al., 2012). These results further demonstrated that leaders with DT personalities amplify the perceptions of victimization of employees in the workplace.

Our results reveal a mediating role of abusive supervision in the link between DT and perceived victimization. The results of both Machiavellianism and psychopathy regarding the mediation analysis were supported and were consistent with prior studies which revealed that organizational heads with cruel minds create an unstable work environment for subordinates (Kiazad et al., 2010). Thus, the findings on the mediating role of abusive supervision in the relationship between a leader’s psychopathic as well as Machiavellianism traits and perceived victimization lend support to the study of Wisse and Sleebos (2016). By implication, leaders who practice psychopathic leadership capitalize on it to inflict pain and displeasure on subordinates in order to clinch on to power. On the contrary, our results concerning the mediating role of abusive supervision in the relationship between narcissism and perceived victimization were found to be insignificant in the sense that narcissists usually display low aggressive behavior, consistent with Baloch et al. (2017). These results further demonstrate that narcissistic leaders are less interested in adopting an abusive leadership style to victimize employees in the workplace, and thus these leaders are social, friendly, and enhance the interest of employees to put up their best in their endeavors.

However, lack of research and inconsistencies (Dadaboyev et al., 2019) in the relationship between DT and individuals’ perceived victimization indicate that there must be some contextual or individual factors which affect this relationship. Considering this, the present study also investigated the moderating role of mindfulness in the relationship between abusive supervision and perceived victimization. The results were consistent with previous studies which revealed a dampening effect of employee mindfulness on abusive supervision, demonstrating that employee mindfulness significantly moderates the relationship between abusive supervision and perceived victimization by neutralizing the effect of the former on workforce outcomes. Our results also supported the findings that when employees have a high degree of mindfulness, they detach themselves from unfavorable work conditions, unlike the employees with a low degree of mindfulness (Zheng and Liu, 2017).

Lastly, we discuss the results of the mediated-moderation relationship among the study constructs. The results indicated a lower impact of DT on perceived victimization when the employees have a high level of mindfulness. Particularly, the results showed that the mediating effect of abusive supervision in the relationship between leaders’ DT traits and employees’ perceived victimization was low for employees possessing high mindfulness as compared to those possessing low mindfulness. A self-regulatory mechanism involving mindfulness plays a central role in diminishing the impact of stressful events that results in decreased perceptions of victimization (Lomas et al., 2019). Thus, our results presented the influential role of mindfulness which helps employees mitigate any adverse work conditions and workplace stressors.

### Theoretical Implications

This research has numerous theoretical implications. Firstly, it adds to the literature on DT and perceived victimization. This research broadens the understanding of leaders’ DT personality, indicating that it affects not only employee outcomes but also how employees perceive victimization in the workplace. As discussed earlier, previous studies on DT have demonstrated its impact on diverse work outcomes such as counterproductive work behaviors (Cohen, 2016) and hard tactics (Jonason et al., 2012). However, to date, the impact of DT on perceived victimization, which has been found to occur in the business sphere (Valentine and Fleischman, 2018), has largely been ignored.

Secondly, while interest in DT and its outcomes has substantiated in recent years (Czarna et al., 2016), little is known about the intermediary factors that can transform DT into either an enhancing or a diminishing factor for perceived victimization. DT has been proven to influence the organizational outcomes or individual outcomes (e.g., employee turnover intention) through affecting the work culture or environment of a workplace (Baloch et al., 2017). Given that perceived victimization takes place more often in an extremely hostile environment (Aquino and Bradfield, 2000), we suggest that the abusive supervision can serve as a mediator between DT and perceived victimization. Yet, thus far, no similar study has focused on the adverse impact of abusive supervision from this perspective.

Tracking the three-phase survey data in this research, it became evident that abusive supervision exerts a critical influence on perceived victimization of employees in the workplace. These findings also show that leaders with DT personality will assuredly encourage a negative work environment in the organization.

Thirdly, our findings reveal the vital role of mindfulness as a self-regulatory individual trait that mitigates the negative consequences of an abusive work environment. For an employee with high mindfulness, it may be more feasible to lower perceptions of victimization regarding hostile and aggressive workplace practices. However, when a potential victimized employee faces a highly abusive work environment, including hostility and retaliation, his/her feelings of workforce-perceived victimization may be significantly expressed (Tepper et al., 2006). Therefore, the effect of abusive supervision on perceptions of victimization is intensely low for employees with high mindfulness. On the other hand, workers with low mindfulness will see abusive supervision as more threatening because they are not capable enough to cope with the negative impact of abusive supervision. Our findings thus reveal that employees’ self-regulatory mechanisms have a significant influence on the types of leadership processes and outcomes.

In this study, a unified moderated pathway assessment approach (Edwards and Lambert, 2007) was used to overcome the research gaps and methodological shortcomings of the survey data collected in previous studies. As such, our research provides useful insights about the link between DT leaders and employees’ perceptions of victimization *via* important mechanisms and assessing its influence by testing a mediated moderation model.

Furthermore, to determine the beneficial effects of the relationship between the independent and dependent variables, we employed a time lag procedure for research design to obtain empirical confirmation of the beneficial effects of DT on perceived victimization by using two types of mechanisms affecting the main relationship.

Finally, our overall holistic mediated moderation model tests provide significant evidence that the degree to which abusive supervision mediates the association between DT and perceived victimization relies on employee mindfulness. Previous studies substantiated that work environments or personal elements are a vital link between DT and its consequences (Czarna et al., 2016). Yet, such studies are silent regarding the specific circumstances whereby the mediating impact of situational or personal elements is intensified or attenuated.

### Practical Implications

A study on the association between the traits of supervisors and the leadership style has significant implications for leadership selection plans. Primarily, our findings indicate that leaders who score high on DT victimize their employees to gain potential advantages. Leaders manifesting high levels of self-interested impulsivity and callousness are particularly not suitable for job positions that entail endurance and considerate acts and social sensitivity, such as the healthcare sector (e.g., nursing). Consequently, we would recommend that entities cogitate screening on DT personality traits when employing people for certain job positions as such personalities could bring about disastrous outcomes. Moreover, our study revealed that leaders with DT personality, through abusive supervision, bring about perceptions of victimization in nurses. This consequence cautions that abusive supervision indeed serves as a threat and is harmful for an entity (Khalid et al., 2018). The healthcare sector should take steps to prevent the detrimental consequences of abusive supervision by using the present study findings to develop programs that are intended to train, educate, and support nurses concerning abusive supervision. Such initiatives could be useful in increasing the understanding and responsiveness of nurses toward abusive supervision, as well as mitigating the potential outcomes that may arise, especially by promoting ways to enforce organizational policies. In other words, there is a need for transparent procedures for nurses to report cases.

Furthermore, attention should be paid to develop employees’ self-regulatory mechanisms in the workplace. For instance, organizations might frequently deliver training agendas to cultivate employee mindfulness in the workplace, which helps employees mitigate the harmful effects of abusive supervision that leads to perceived victimization. Since changing a leader’s behavior is extremely difficult (Karthikeyan and Joy, 2018) and abusive supervision is a common phenomenon in management (Tepper, 2000), we advocate that organizations should encourage mindfulness as a valuable resource to cope with destructive leadership, i.e., abusive supervision. Studies have revealed that mindfulness can indeed be developed and improved by training strategies (Brown and Ryan, 2003). Therefore, institutions should consider educating leaders as well as the workforce about self-regulatory capabilities such as mindfulness since it is considered as a key ability that might allow individuals to accomplish career growth in the workplace (Glomb et al., 2011; Lomas et al., 2019). We also encourage employees to focus their energy on developing mindfulness on their own, thereby protecting themselves from the harmful effects of unpleasant experiences like abusive supervision.

### Limitations and Future Studies

Our study has some limitations which can offer an avenue of research for future studies. First, the data were gathered only from the healthcare sector of one country, Pakistan. Hence, future studies should target other sectors and cultures for more generalized findings. Moreover, our research emphasizes on abusive supervision as a mediating role in the DT-perceived victimization association and mindfulness as a moderator in the relationship between abusive supervision and perceived victimization. A promising suggestion for future studies is to investigate other mediating and moderating mechanisms that can elucidate the DT-perceived victimization association. Furthermore, we employed the Dirty Dozen Scale developed by Jonason and Webster (2010) to assess DT. The detailed measures may be helpful if unnecessary items are eliminated (Czarna et al., 2016). Thus, future investigations should use other scales, such as the SD3 of Jones and Paulhus (2014) or the complete scale of DT. Lastly, the current study relied on subordinates’ responses. Future studies could investigate DT tendencies from the perspectives of other persons such as leaders, customers, auxiliary staff, and other fellow supervisors.

## Conclusion

Drawing upon the social exchange theory and integrating it with the self-regulatory approach, our research uncovered when and how leaders with DT personality victimize the workforce. Our results indicate that abusive supervision serves as a mediating factor underlying the link between DT and perceived victimization. Furthermore, mindfulness moderates the detrimental impacts of abusive supervision, which affects the DT-perceived victimization association in the workplace.

Our study also has implications for literature on DT, abusive supervision, mindfulness, perceived victimization, and the social exchange theory. We expect that our study will inspire further efforts to advance our understanding in this field.

## Data Availability Statement

The raw data supporting the conclusions of this article will be made available by the authors, without undue reservation, to any qualified researcher.

## Ethics Statement

The studies involving human participants were reviewed and approved by the Ethics Committee of Jiangsu University. The patients/participants provided their written informed consent to participate in this study. Written informed consent was obtained from the individual(s) for the publication of any potentially identifiable images or data included in this article.

## Author Contributions

HK, MZ, and SS conceptualized the study objectives and framework. HK designed the methodology and conducted the data analysis. MK reviewed the manuscript. All authors contributed to the article and approved the submitted version.

## Conflict of Interest

The authors declare that the research was conducted in the absence of any commercial or financial relationships that could be construed as a potential conflict of interest.
